# Morphologic Differentiation of Viruses beyond the Family Level

**DOI:** 10.3390/v6124902

**Published:** 2014-12-09

**Authors:** Cynthia S. Goldsmith

**Affiliations:** Infectious Diseases Pathology Branch, Division of High-Consequence Pathogens and Pathology, Centers for Disease Control and Prevention (CDC), Atlanta, GA 30333, USA; E-Mail: cgoldsmith@cdc.gov; Tel.: +1-404-639-3306

**Keywords:** virus, electron microscopy, *Poxviridae*, *Reoviridae*, *Retroviridae*, *Herpesviridae*, *Filoviridae*, *Bunyaviridae*

## Abstract

Electron microscopy has been instrumental in the identification of viruses by being able to characterize a virus to the family level. There are a few cases where morphologic or morphogenesis factors can be used to differentiate further, to the genus level. These include viruses in the families *Poxviridae*, *Reoviridae*, *Retroviridae*, *Herpesviridae*, *Filoviridae*, and *Bunyaviridae*.

## 1. Introduction

The electron microscope has been a powerful tool in the characterization of viruses. First constructed by Ernst Ruska and Max Knoll in the early 1930s, the electron microscope was soon used in the visualization of an orthopoxvirus, mouse ectromelia virus [1]. The potential for using this newly developed instrument for the understanding of the ultrastructure of viruses and other pathogens was quickly recognized by Helmut Ruska, a physician and the brother of Ernst [2,3]. In the ensuing years, many viruses were recognized by electron microscopy (EM), and were characterized by features such as size, shape, the appearance of the capsid, presence or absence of an envelope, surface projections, and method and site of morphogenesis. These traits can identify the virus to a family level, since, in general, the morphologic features within a given family are the same. Interestingly, the first report of the International Committee on Taxonomy of Viruses (ICTV) released in 1971 recognized only two families of viruses, *Papovaviridae* and *Picornaviridae*. All other recognized viruses were listed as “Unassigned” and mostly categorized as genera [4]. This was at a time when examining virus ultrastructure by EM permitted the grouping of viruses on a morphological basis [5,6], and in the future EM played a critical role in the taxonomic classification of viruses [7].

Electron microscopists typically use negative stain and thin section EM for diagnostic virology. Negative stain EM entails adsorbing a biological fluid (e.g., cell culture supernatant, urine, cerebral spinal fluid, *etc.*) onto an EM grid coated with a plastic film. The viruses that adhere to the grid are stained with a heavy metal which pools around the viruses, giving them an appearance as seen in a photographic negative, *i.e.*, a light specimen against a dark background. Proteins on the surface of the nucleocapsid or envelope become apparent, and which allows for a morphologic differentiation among the different virus families [8]. In thin section EM, tissues or tissue culture cells are embedded in an epoxy resin and cut into ultra-thin sections (e.g., 70–90 nm). This allows the electron microscopist to examine cells and viruses in a cross-sectional view. The virus family can be determined by evaluating the morphogenesis of the virus by looking at the site of assembly, the location of envelope acquisition, and other clues that may be offered by replication complexes [9].

There are a few examples where either thin section or negative stain EM, or both, can go beyond the family level classification. Currently, there are 103 recognized virus families, of which 22 infect humans. This review will discuss six examples where morphologic features allow for diagnosis of a virus not just to the family level, but to the genus level.

## 2. Virus Families

### 2.1. Poxviridae

Poxviruses are the largest and most complex of the viruses causing human disease. The most infamous would be variola virus, the causative agent of smallpox, which was eradicated by a concerted global effort overseen by the World Health Organization; the last naturally occurring case was in 1977. Infections with a poxvirus will produce pock(s), or pustule(s), on the skin and also internally on visceral organs with some species. The genera of poxviruses that can cause human disease include *Orthopoxvirus*, *Parapoxvirus*, *Molluscipoxvirus*, and *Yatapoxvirus*.

Although poxviruses contain DNA, the DNA replication and virus assembly do not take place in the nucleus but rather in the cytoplasm. Virus factories, or virosomes, are created and nascent crescents are formed and engulf the unit genome. Immature particles are spherical, but condense down to a dumbbell-shaped intracellular mature virus (IMV). The IMV is engulfed by Golgi vesicles, migrates to the cell surface or into microvilli and fuses with the cell membrane, releasing a particle wrapped in a single membrane known as an enveloped extracellular virus (EEV).

**Figure 1:**
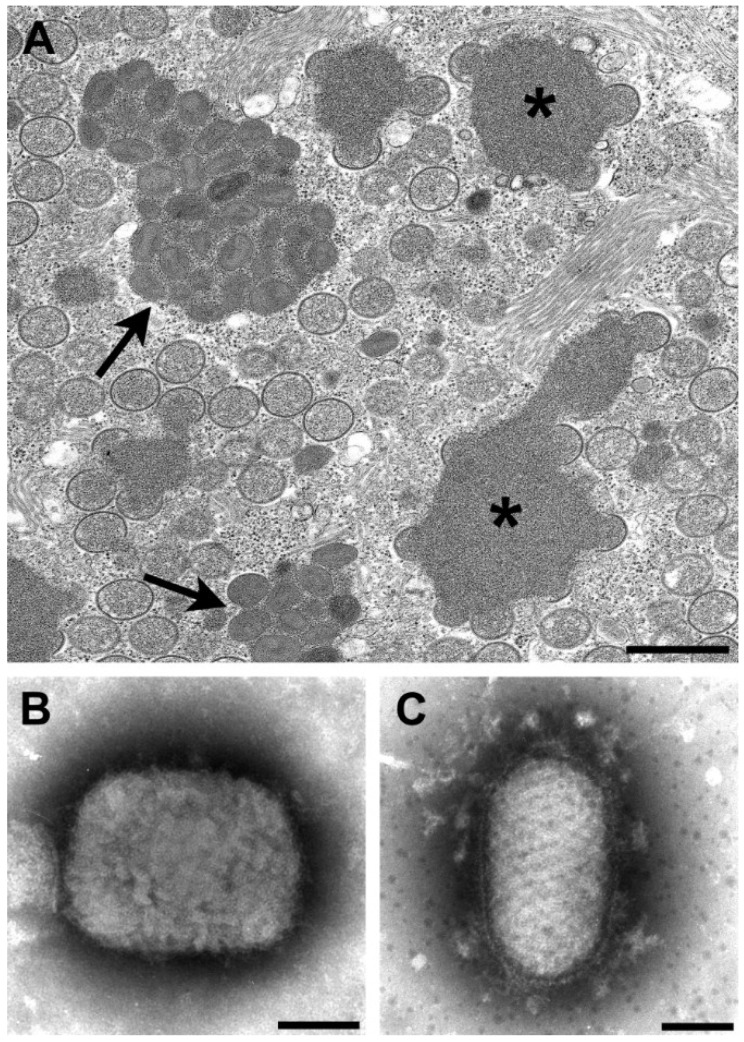
(**A**) Thin section image of raccoonpox, showing viral factories (*) and A-type inclusions (arrows). Bar, 500 nm; (**B**) Negative stain image of a clinical sample of monkeypox virus (genus *Orthopoxvirus*). Bar, 100 nm; (**C**) Negative stain image of a clinical sample of orf virus (genus *Parapoxvirus*). Bar, 100 nm.

By thin section EM, in addition to the viral factories, some poxviruses have other cytoplasmic structures know as acidophilic-type inclusions (A-type inclusions) consisting of a matrix containing the A-type inclusion protein and other proteins, with occluded intracellular mature particles [10,11] (Figure 1A). Virus species with these inclusions include cowpox, ectromelia, raccoonpox, skunkpox, volepox, and fowlpox viruses.

The viruses in the genera *Orthopoxvirus* and *Parapoxvirus* can be distinguished by negative stain EM (Figure 1B,C). Orthopoxviruses are rectangular, approximately 225 × 300 nm in size and have a surface pattern of short, whorled filaments. On the other hand, parapoxviruses are oval, average only about 150 × 200 nm in size, and have a crisscross filamentous surface pattern. Unfortunately, these two genera cannot be definitively differentiated by thin section EM.

### 2.2. Reoviridae

Reoviruses derive their name from **R**espiratory **E**nteric **O**rphan viruses. This paper will describe viruses that are members of the genera *Orthoreovirus* and *Rotavirus*. Orthoreoviruses are in the subfamily *Spinoreovirinae* and contain large spikes or turrets at the 12 icosahedral vertices of the core particle, while rotaviruses are in the subfamily *Sedoreovirinae* and do not have large surface projections on the core particles [12].

**Figure 2:**
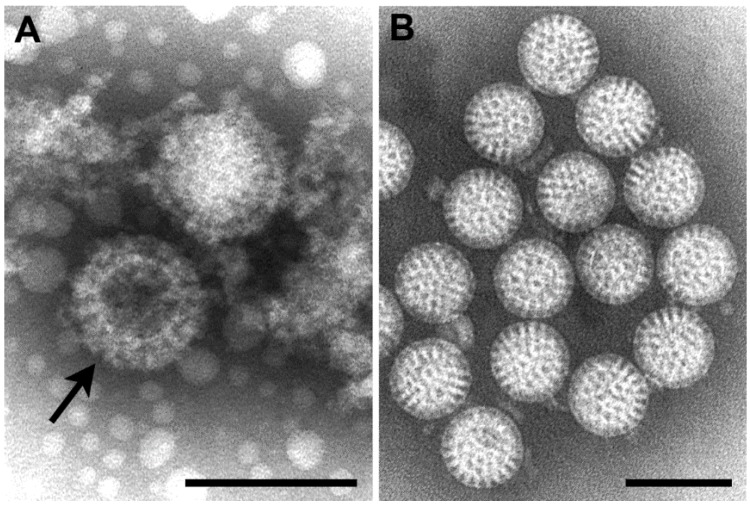
(**A**) Negative stain EM image of orthoreovirus particles, with a stain-penetrated particle (arrow) showing the double capsid layers; (**B**) Negative stain EM image of rotavirus particles. Bars, 100 nm. (Figure B, courtesy of Charles D. Humphrey, Centers for Disease Control and Prevention, Atlanta, GA, USA.)

Orthoreoviruses are spread by the respiratory or fecal-oral routes. The genome consists of 10 segments of linear double-stranded RNA. By negative stain EM, virions are approximately 85 nm in diameter, are roughly spherical, and possess a double-layered protein capsid (Figure 2A).

Rotaviruses are the cause of severe diarrheal disease in infants and young children, and were first recognized by EM in 1973 [13]. The genome is composed of 11 segments of linear double-stranded RNA. By negative stain EM, virus particles are 70 nm in diameter and are constructed of three concentric protein layers. Virions have a wheel-like appearance (*rota* is Latin for “wheel”) with a sharp definition of the outer margin (Figure 2B).

### 2.3. Retroviridae

The retroviruses are divided into two subfamilies. The genera in the subfamily *Orthoretrovirinae* that can infect humans are *Alpharetrovirus*, *Betaretrovirus*, *Deltaretrovirus*, *Gammaretrovirus*, and *Lentivirus*. *Spumavirus* is the only genus in the subfamily *Spumaretrovirinae*. The viral genome for all but the spumaviruses consists of a dimer of positive-sense, single-stranded RNA held together by hydrogen bonds. Spumaviruses contain double-stranded DNA. All retroviruses use the enzyme reverse transcriptase to transcribe an RNA template into complementary DNA.

**Figure 3:**
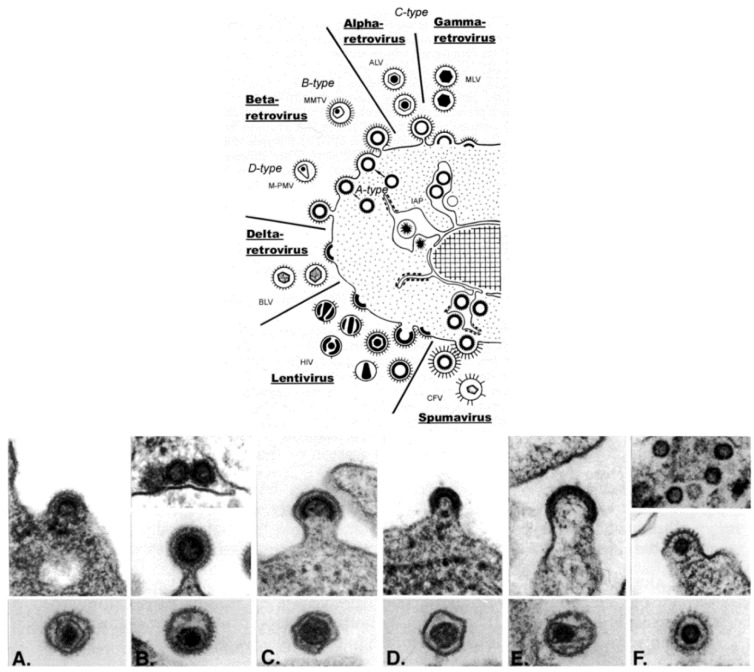
(**Top**) schematic diagram of the morphogenesis of the members of the family *Retroviridae*; (**Bottom**) budding profiles and mature virions. (**A**) Avian leukosis virus (genus *Alpharetrovirus*); (**B**) Mouse mammary tumor virus (genus *Betaretrovirus*); (**C**) Murine leukemia virus (genus *Gammaretrovirus*); (**D**) Bovine leukemia virus (*Deltaretrovirus*); (**E**) Human immunodeficiency virus 1 (genus *Lentivirus*); (**F**) Simian foamy virus (genus *Spumavirus*). (Top, reproduced with permission from Reference [14]. Copyright 2002 Kluwer Academic/Plenum Publishers. Bottom, reproduced with permission from Reference [17]. Copyright 1997 Cold Spring Harbor Laboratory.

*Retroviridae* is, morphologically, a multi-faceted family of viruses. As illustrated in a drawing that appeared in a publication by Gelderblom and Boller [14] (Figure 3, top), in thin section EM, members of the family can be assigned to a particular genus based on the morphogenesis of the virus and on the appearance of mature virus particles [15,16] (Figure 3, bottom).

The genera *Alpharetrovirus* and *Gammaretrovirus*, such as avian leukosis virus (ALV) and murine leukemia virus (MLV), respectively, were previously known as C-type particles; the cores form concomitantly with budding, and are centered in the middle of mature particles. Viruses previously known as B-type (such as mouse mammary tumor virus (MMTV)) and D-type (such as Mason-Pfizer monkey virus (M-PMV)) are now part of the genus *Betaretrovirus*. Both types are formed by envelopment of pre-formed cores, which are known as A-type particles, and mature into eccentrically located cores surrounded by the viral envelope. The viruses in the genus *Deltaretrovirus*, such as bovine leukemia virus (BLV), have a crescent-shaped budding profile which is composed of an electron-dense nucleoid and the nascent viral envelope. The cores of the mature virions are somewhat pleomorphic and fairly homogeneous, and there is often an electron-lucent space between the core and the envelope. Human immunodeficiency virus (HIV) is an example of the genus *Lentivirus*, and these viruses also have a crescent-shaped budding profile, which can be released from the cell to form a doughnut-shaped particle. The nucleoid then condenses into an electron-dense core that is cone-shaped, but can appear as a rod. The viruses in the genus *Spumavirus*, such as chimpanzee foamy virus (CFV), are seen as contaminants in some cell cultures derived from animal organs. Pre-formed cores are enveloped at cellular membranes or the plasma membrane, and the cores do not condense.

### 2.4. Herpesviridae

**Figure 4:**
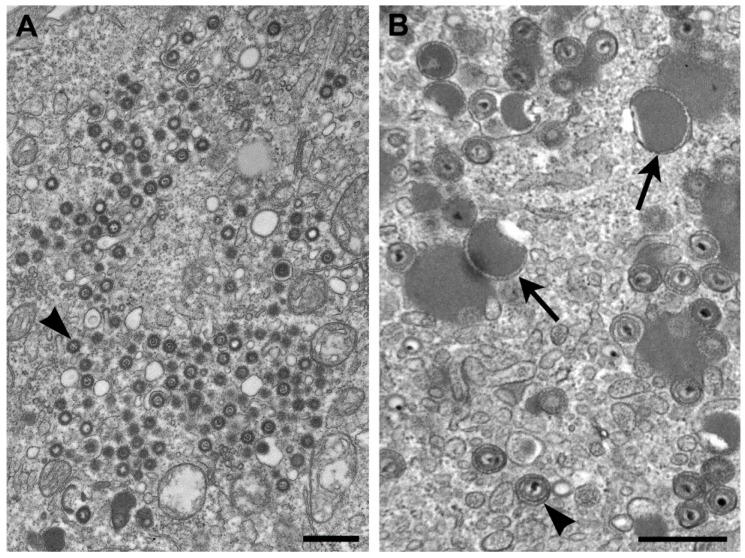
(**A**) Thin section EM image of the cytoplasm of a cell infected with human herpesvirus 7. Note that although there are nucleocapsids surrounded by tegument (arrowhead), there are no dense bodies; (**B**) Cell infected with simian CMV, with dense bodies (arrows) and virus particles (arrowhead) in the cytoplasm. Bars, 500 nm. (Figure B, courtesy of Sara E. Miller, Duke University Medical Center, Durham, NC, USA.)

The genus *Cytomegalovirus* (CMV) is within the subfamily *Betaherpesvirinae*, and infection with these viruses typically results in an increase in cell volume (cytomeglia). CMV-infected cells have nuclear inclusions, characteristic of herpesviruses, but also have cytoplasmic inclusions. The genomes of herpesviruses in general are composed of linear, double-stranded DNA. Replication takes place in the nucleus where nucleocapsids are formed, are surrounded by an “inner” tegument, and travel to the cytoplasm by budding upon the inner nuclear membrane, passing through the perinuclear space, and fusing with the outer nuclear membrane to egress to the cytoplasm. Additional tegument proteins attach to the capsid, either within the cytosol and/or at the future envelopment site on the membranes of the Golgi complex [18] (Figure 4A).

Unlike the other members of the family *Herpesviridae*, the cytoplasm in cells infected by CMV contains numerous aggregations of enveloped tegument proteins that lack capsids (Figure 4B). These are known as dense bodies [19], which are highly immunogenic and have been proposed as CMV vaccine candidates since they induce both humoral and cellular immune responses [20].

### 2.5. Filoviridae

This family includes *Ebolavirus* and *Marburgvirus* genera and the recently recognized *Cuevavirus* genus, which is not known to cause human disease. Filoviruses are nonsegmented, negative-sense, single-stranded RNA viruses. Virions are pleomorphic, appearing as long filamentous particles, but also as branched, 6-shaped, U-shaped, or circular particles.

The viruses in the genera *Ebolavirus* and *Marburgvirus* are well-known as being some of the deadliest known viruses, with case fatality rates for Ebola virus reported at 50% to 90%, and rates for Marburg virus at 24% to 88%. Early symptoms for both diseases include sudden onset of fever, headaches, weakness, muscle pains, and a sore throat. As the diseases progress, additional symptoms such as vomiting, diarrhea, impaired kidney and liver function, and sometimes a rash and internal and external bleeding may develop.

**Figure 5:**
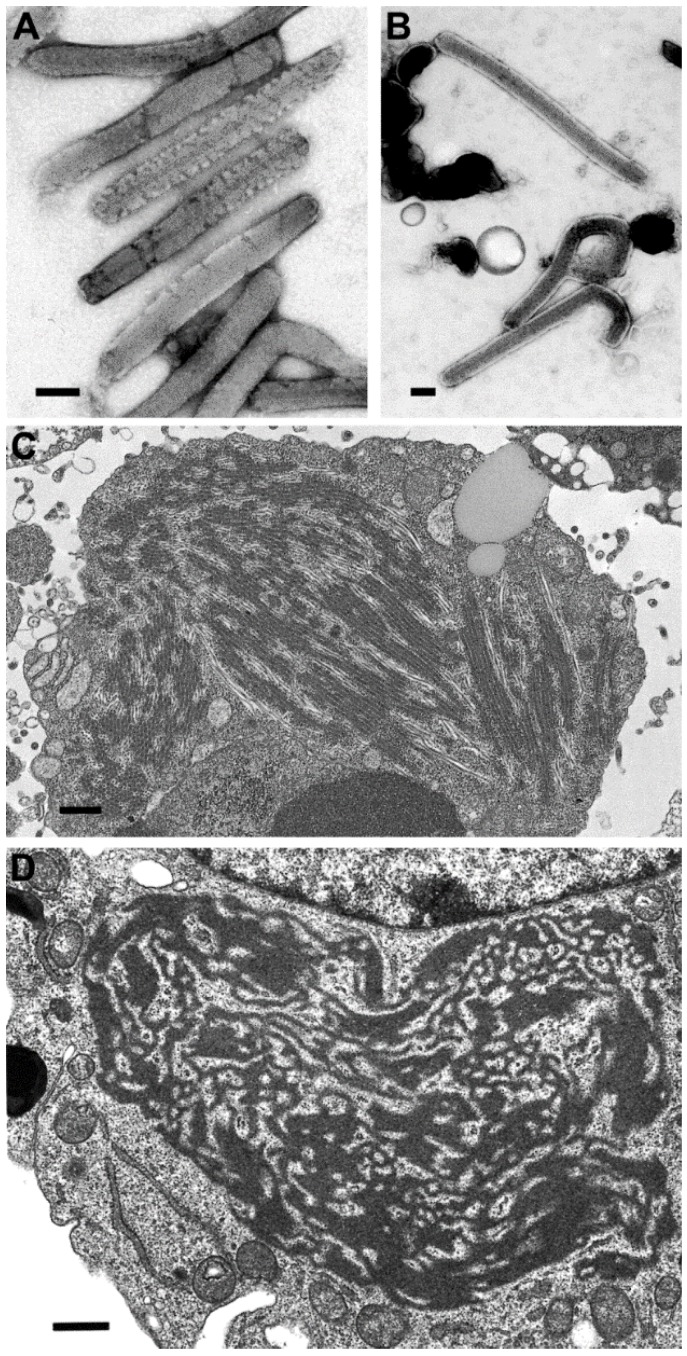
Negative stain images of Marburg virus (**A**) and Ebola virus (**B**), illustrating that Ebola virus has a longer length than Marburg virus. Bars, 100 nm; (**C**) Thin section image of a large inclusion in an Ebola virus-infected cell. Bar, 500 nm; (**D**) Intermediate inclusion in the cytoplasm of a Marburg virus-infected cell. Bar, 580 nm. (Figure A, courtesy of Russell Regnery, Centers for Disease Control and Prevention; Figure D, courtesy of Thomas Geisbert, United States Army Medical Research Institute of Infectious Diseases, Frederick, MD, USA.)

There have been differences reported in the morphologic features of Ebola and Marburg viruses. First, the lengths of the virus particles of the two genera vary, although there have been different lengths reported. For instance, there have been reports of 665 nm, 790 nm, and 860 nm lengths for Marburg viru*s*, and 805 nm, 970 nm, and 1200 nm for Ebola virus, but clearly Ebola viruses are longer [21,22,23] (Figure 5A,B). A second difference is found in the ultrastructure of the intermediate inclusion of the viruses. Ebola virus inclusions have distinct preformed nucleocapsids mixed with lighter-staining matrix material and, at times, naked nucleocapsids are present (Figure 5C). The inclusions in Marburg virus infections begin with light-staining nascent viral material, which increases in electron density as the infection progresses, and has 45–60 nm spheres of inclusion material surrounding the inclusion. Later, the intermediate inclusions show a dispersal of material and a loss of the spheres [23,24] (Figure 5D).

### 2.6. Bunyaviridae

The family *Bunyaviridae* contains four genera that can infect humans—*Orthobunyavirus*, *Nairovirus*, *Phlebovirus*, and *Hantavirus*. Viruses contain single-stranded RNA with three RNA segments that are negative sense, with the exception of phleboviruses which have one ambisense segment. These are zoonotic viruses, where each virus is associated with a specific vector or natural reservoir, including mosquitoes, ticks, sand flies, and rodents. Patients will usually have a hemorrhagic syndrome which is characterized by fever, increased capillary permeability, leukopenia and thrombocytopenia.

**Figure 6:**
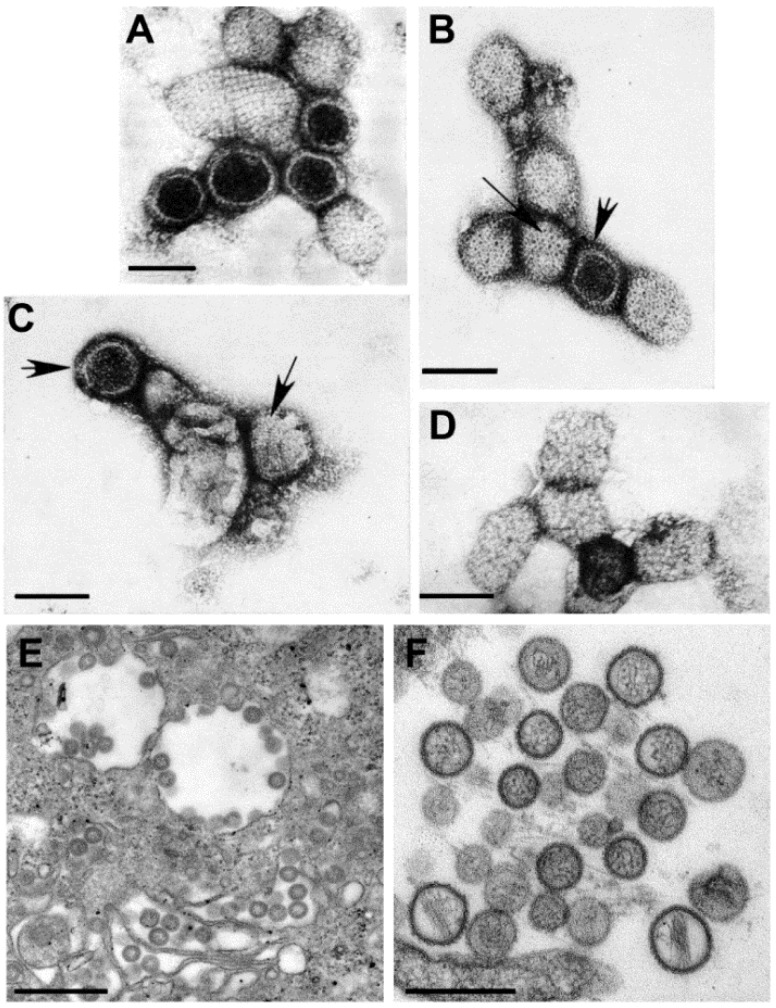
(**A**–**D**) Negative stain EM preparations. (**A**) Hantaan virus particles (genus *Hantavirus*) showing a grid-like surface pattern. Bar, 100 nm; (**B**) Rift Valley fever virus (genus *Phlebovirus*) exhibiting subunits with a central hole (long arrow) and showing the regularly spaced subunits (short arrow). Bar, 100 nm; (**C**) Crimean-Congo hemorrhagic fever virus (genus *Nairovirus*) having small surface subunits (long arrow) which appear as a peripheral fringe (short arrow). Bar, 100 nm; (**D**) Anhemi virus (genus *Orthobunyavirus*) with knob-like surface structures. Bar, 100 nm; (**E**,**F**) Thin section EM preparations; (**E**) Virions have moderately dense centers and accumulate in the cisternae of the Golgi complex of a Rift Valley fever virus-infected cell. Bar, 500 nm; (**F**) Extracellular Sin Nombre virus particles (genus *Hantavirus*) have a variety of sizes and internal cores composed of thin thread-like material. Bar, 500 nm. (Figures A–D, courtesy of Mary Lane Martin, Centers for Disease Control and Prevention; Figure E, courtesy of Frederick A. Murphy, Centers for Disease Control and Prevention.)

In negative stain preparations, Martin, *et al.* [25] were able to distinguish among the genera of the family, according to the surface arrangement of structural units. Hantaviruses have the most distinct structure, where the surface units are arranged in a square, grid-like pattern (Figure 6A). Phleboviruses, which have recently incorporated the genus *Uukuvirus*, have a surface structure formed of subunits with a distinct central hole (Figure 6B). The surface of nairoviruses have very small morphologic units (Figure 6C), and orthobunyaviruses exhibit either knob-like surface units or are indistinct (Figure 6D).

By thin section EM, the virions of most bunyaviruses have a fairly homogeneous core and small spikes on the virus surface are visible in some preparations. The viruses mature by budding upon the membranes of the Golgi complex and can accumulate in the Golgi cisternae or extracellularly [26] (Figure 6E). The viruses in the genus *Hantavirus*, however, have a core consisting of thin threads of ribonucleoproteins (Figure 6F). In addition, in cell cultures hantaviruses can mature by budding from the plasma membranes of infected cells, and are associated with tubular projections [27,28].

## 3. Discussion

EM can be an important factor in the initial diagnosis of a viral infection, in conjunction with serological, pathological, and molecular assays. At times, EM has provided identification of a virus when these other methods were unsuccessful [29]. EM is an unbiased assay, in that there is no need for specific antibodies or molecular probes; instead, what is visible at the EM level can be detected and categorized. However, it takes an experienced electron microscopist trained in virology to make the morphologic distinctions.

Other tools are available that can be used to determine the genus of a virus. Biochemical methods consist of hemagglutination, hemagglutination inhibition, virus neutralization, and immunohistochemistry. Molecular assays include PCR, sequencing, next generation sequencing, and *in situ* hybridization. These techniques can be used to verify, or in tandem with, the EM examples discussed here to diagnose a virus to the genus and/or species level.

The analysis of virus structure has advanced greatly over the years. Negative stain EM and X-ray diffraction have long been used in the analysis of virus ultrastructure. Thin section EM is critical in the study of viral morphogenesis, and may be preferred over negative stain EM if the viruses are highly cell-associated. CryoEM using single particle analysis and 3D tomography has greatly furthered our knowledge on the architecture of viruses [30]. In addition, scanning EM and atomic force microscopy can add to the understanding of the structural biology of viruses [31,32]. These techniques, along with future technologies, will continue to improve our ultrastructural understanding of viruses.

In summary, EM identification of a virus in a clinical sample or a virus isolate can be instrumental for a diagnosis. Taxonomic classification for viruses by using EM has generally been at the family level, with a few exceptions. The ability of electron microscopists to discern the genus of a virus, as described in this paper, can be of great value to the clinician or to the laboratorian using cell culture for virus isolation.
